# Tibiofemoral Contact Forces Influence Intraoperative Kinematic Pivot Pattern Dependent on Posterior Cruciate Ligament Resection in Primary Total Knee Arthroplasty

**DOI:** 10.5435/JAAOSGlobal-D-22-00033

**Published:** 2022-04-05

**Authors:** Evan R. Deckard, Mary Ziemba-Davis, R. Michael Meneghini

**Affiliations:** From the Indiana University School of Medicine, Department of Orthopaedic Surgery, Indianapolis, IN (Deckard, and Dr. Meneghini), and the Indiana University Health Physicians, IU Health Hip & Knee Center, Fishers, IN (Ziemba-Davis, and Dr. Meneghini).

## Abstract

**Background:**

Optimizing knee kinematics has the potential to increase patient satisfaction with total knee arthroplasty (TKA); however the ability to enact a particular kinematic pattern is variable and inconsistent. The purpose of this study was to determine whether intraoperative contact forces were predictive and can potentially drive a particular kinematic pivot pattern.

**Methods:**

All TKAs used sensor-embedded tibial trials to intraoperatively measure medial and lateral compartment forces, and the associated condylar contact points were used to calculate kinematic pivot patterns between preceding flexion angles.

**Results:**

After exclusions, 157 TKAs were analyzed. For posterior cruciate ligament–intact TKAs, no predictors of lateral pivot were identified in early flexion; however, increased medial compartment force and increased lateral compartment force were predictors of medial and lateral pivots for mid and late flexion, respectively (*P* ≤ 0.037). For posterior cruciate ligament–resected TKAs, increased lateral compartment force was a predictor of lateral pivot in early and midflexion (*P* ≤ 0.031) but not late flexion.

**Conclusion:**

The tibiofemoral compartment with greater contact force exhibited less anteroposterior translation at certain flexion ranges and correlated with kinematic pivot patterns. This information may benefit surgeons who are attempting to facilitate a particular kinematic pattern. Further research is recommended to confirm that intraoperative kinematics correlate with weight-bearing postoperative kinematics and clinical outcomes.

Despite excellent long-term survivorship and outcomes after total knee arthroplasty (TKA),^1,2^ patient satisfaction stubbornly lags behind that of total hip arthroplasty, with approximately 20% of unsatisfied patients. Unsatisfied patients experience pain, functional limitations, and describe an unnatural feel to the knee.^3^ To help close the gap on the subset of unsatisfied patients, there is increased interest in knee kinematics before and after TKA related to kinematic pivot patterns, which may help optimize patient-reported outcomes. Traditionally, it was hypothesized that the optimal kinematic pivot pattern was predominantly medial through the range of motion.^4,5^ However, a modern understanding of kinematic pivot patterns in healthy knees and post-TKA knees may be nuanced with a more accurate description of a dual-pivot kinematic pivot pattern characterized by a lateral pivot in early flexion, which transitions to a medial pivot in mid to late flexion.^6,7,8,9^ Recently, this dual-pivot kinematic pivot pattern has shown superior patient-reported outcomes related to satisfaction and patients reporting their knee to always feel normal at 1-year after TKA compared with various other patterns.^10^ In contrast, strictly medial pivot designs show comparable but not significant advantages in patient-reported outcomes compared with non–dual-pivot TKA designs.^11,12,13,14,15^

In addition, gap balancing has become a popular technique for obtaining a symmetric flexion space in TKA. It is reported that unbalanced medial and lateral collateral ligaments after TKA can lead to decreased stability, functional limitations, and even deleterious long-term implant wear and loosening.^16^ Real-time feedback during TKA is now possible to guide soft-tissue release and balance using trial tibial inserts to quantify peak loading forces in the tibiofemoral compartments.^17,18^ However, despite acceptance and adoption of this attractive sensor technology, evidence regarding the influence of compartment balance on satisfaction remains equivocal.^19-21^ Although this newly available technology is intriguing and understanding of knee kinematics is improving, the ideal target for tibiofemoral compartment balance remains elusive. Furthermore, whether balanced collateral ligaments after TKA can produce a specific kinematic pivot pattern remains unknown. Because surgeons have some control over soft-tissue balance through bone cuts and soft-tissue release, understanding whether differences in compartment forces via gap balancing can drive a particular kinematic pivot pattern would benefit TKA surgeons.

The purpose of this study was to determine whether medial and lateral compartment forces measured intraoperatively with trial tibial insert sensors facilitate a particular kinematic pivot pattern during passive knee motion. The null hypothesis was that intraoperative compartment forces would not dictate kinematic pivot patterns during passive knee motion.

## Methods

**Figure 1:**
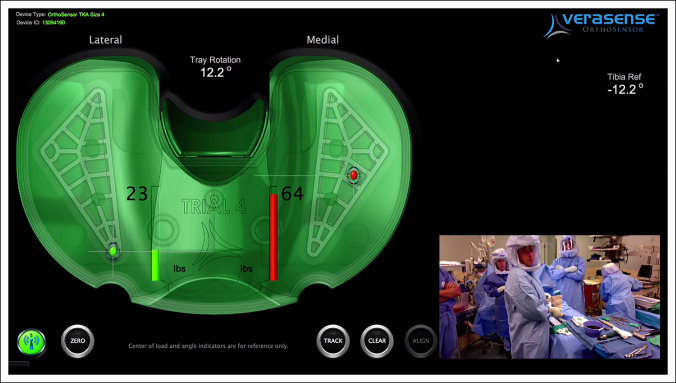
Image showing graphic user interface for the trial tibial insert. Green and red circles represent the condylar contact points. Contact forces are displayed numerically next to its corresponding compartment: a lateral force of 23 lbs and a medial force of 64 lbs.

A retrospective review of a prospectively collected database was conducted on 216 consecutive primary TKAs in which trial tibial inserts were used (Verasense; OrthoSensor). All procedures were performed between April 2013 and January 2014 by two board-certified arthroplasty surgeons at a single institution. The trial tibial inserts were equipped with force sensors to measure tibiofemoral forces (in lbs) in the medial and lateral compartments following standard balancing techniques based on tactile surgeon judgment. The intraoperative force-sensing device comprises a single-use trial tibial insert with a graphic user interface (Figure 1).

Institutional review board approval was obtained for this study, which included patients undergoing primary TKA for osteoarthritis, osteonecrosis, inflammatory arthritis, or posttraumatic arthritis. Patients with a prior TKA or ligament insufficiencies and those requiring posterolateral reconstructions, osteotomies, or repair of tibial plateau fractures were not included in the study. Fifty-eight cases of the original cohort were excluded from analysis due to unavailability of the required size of the tibial insert device (31), device malfunction (18), atypical implant (5), and surgeries performed at a nonstudy hospital (4).

### Surgical Technique

A median parapatellar approach was used for all procedures. Standard coronal plane tibial and femoral bone cuts were made with computer-aided navigation. One knee arthroplasty system (Triathlon Total Knee System; Stryker Orthopaedics) was used in all patients. One surgeon used cruciate-retaining femoral components with cruciate retaining (CR) or cruciate substituting (CS)/anterior lipped inserts, and one surgeon used PS femoral components with PS inserts for most patients, but used CR/CS anterior lipped inserts if patient anatomy dictated a small size to minimize chance of femoral fracture with the cam-post preparation. Medial and lateral compartments were assessed for symmetry and balance in flexion and extension using standard techniques of subjective surgeon tactile sensation. Measured resection and subsequent soft-tissue releases were used to obtain symmetric and balanced flexion and extension gaps along with mediolateral balance. The patella was then prepared, the trial patella was inserted, and patellofemoral tracking was assessed. All trial components were then removed except the standard trial tibial insert, which was securely pegged into place in the correct rotation. The tibia was prepared and bony surfaces were irrigated and cleansed, and the final implants were implanted along with the appropriately sized trial tibial insert. No further ligament releases or balancing techniques were allowed at this point in the procedure so that outcomes would be correlated with the most accurate intraoperative tibiofemoral contact points and compartment force measurements. Measurements were obtained using a uniform data collection protocol with the patella located in the trochlear groove and the retinaculum closed with towel clips, as has been described in multiple studies to provide more accurate measurements.^22,23^

### Intraoperative Compartment Force Measurements

Three medial and three lateral compartment force measurements were made for each patient at full extension, at 45° and 90° of flexion, and at terminal flexion. The three medial measurements and the three lateral measurements were averaged to derive the best estimate of compartment forces across each flexion arc. Best estimate force measurements for early flexion (0° to 45°), midflexion (45° to 90°), and late flexion (90° to terminal flexion) were then averaged to obtain medial and lateral compartment forces across each of the three flexion arcs. Delta compartment forces (medial minus lateral) also were calculated for each flexion arc. Negative difference values reflect greater lateral forces, and positive values reflect greater medial forces. One patient with excessive lateral compartment forces at all flexion angles was identified as an outlier and subsequently was removed from analysis.

### Intraoperative Tibiofemoral Loading Contact Point Measurements

**Figure 2:**
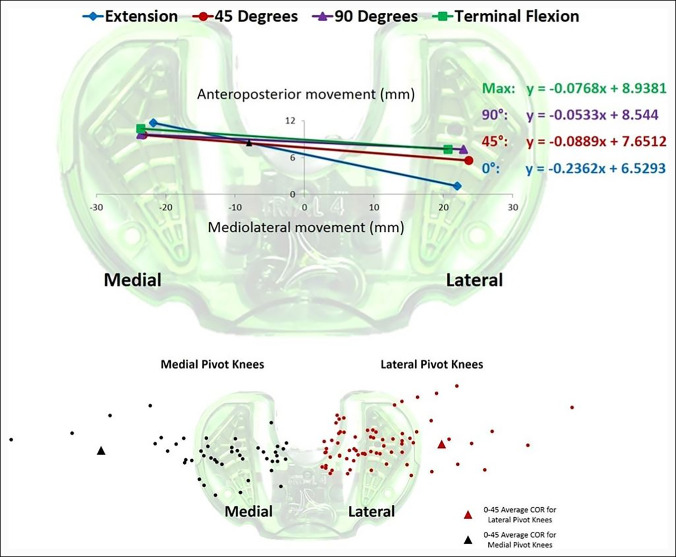
Top, Condylar contact points at each flexion angle. The COR (indicated by a black triangle for early flexion) was calculated by equating the lines created by the 0° and 45° condylar contact points. This knee would be classified as a medial pivot for early flexion. Bottom, Calculated COR values for early flexion (0° to 45°) for both medial pivot knees and lateral pivot knees using the 5- to 1,000-mm definition.

Throughout each procedure, live video feedback of the trial tibial insert was recorded in parallel with the surgeon maneuvering the knee throughout the range of motion (0°, 45°, 90°, and terminal flexion). For each TKA, compartment force measurements were recorded from the video feed. An image of the condylar contact points was recorded from the graphic user interface of the device at each flexion angle (Figure 2).

From these images of the condylar contact points collected at the four flexion angles, an axial center of rotation (COR) was calculated based on the position of the condylar contact points between two given flexion angles. The COR was operationally defined as the intersection point between two preceding flexion angle lines made by the condylar contact points. The medial and lateral condylar contact points at each flexion arc (extension, 45°, 90°, and terminal flexion) allowed a line to be produced based on where the contact points were located. An equation for each line was determined in the algebraic slope-intercept format (*y* = m*x* + b) within Microsoft Excel. Equating two line equations between preceding flexion angles and solving for *x* produced the intersection point and therefore the COR between those two flexion angles similar to methodology used by Dennis et al.^5^

COR values were then used to determine whether the kinematic pivot pattern between the two flexion angles was medial or lateral based on their location with reference to the medial and lateral compartments. A 5-mm to 1,000-mm interval was used as the range within which to specify the kinematic pivot pattern. If the COR was located in the medial compartment between 5 mm and 1,000 mm, the kinematic pattern was determined to be a medial pivot knee between the two distinct flexion angles. If the COR was located in the lateral compartment between −5 and −1,000 mm, the kinematic pattern was determined to be a lateral pivot knee between the two distinct flexion angles. If the COR was less than 5 mm or greater than −5 mm, it was considered a central pivot. If the COR was greater than 1,000 mm or less than −1,000 mm, it was considered a translation of the implant due to the COR value not allowing a detectable pivot pattern and therefore sliding instead of rotating. This methodology has been described and previously published.^10,11^ Patients with central or translational pivot patterns across any flexion arc were excluded from statistical analyses to strictly focus on medial and lateral pivot knees.

### Tibiofemoral Alignment

Tibiofemoral alignment was measured for all patients with a standardized measurement protocol consistent with existing peer-reviewed literature.^24^ Patients received short knee radiographs at preoperative and postoperative clinic visits per standard of care. Radiography was performed by a trained and certified orthopaedic radiologist using standard and accepted techniques. Preoperative and postoperative weight-bearing AP view radiographs were accessed and measured in the Synapse software system (Fujifilm). If multiple images were available, the image with the best quality was used. Preoperative and postoperative tibiofemoral angles were assessed by measuring and bisecting two sets of points based on femoral and tibial landmarks. Calibration was unnecessary due to distances being unrelated to the angle being measured.

The distal-most aspect of the femoral condyle was located and tracked 60 mm proximally to the cortical edge of the femur. The same technique was performed on the medial and lateral sides to create the first set of femoral points. The second set of femoral points was created by measuring 30 mm proximally from the first set of points along the cortical edges (90 mm from the distal-most condyle). These two sets of femoral points were then bisected to create the femoral line.

The tibial line was created in similar methodology to the femoral line. The proximal-most aspect of the tibia was identified. A 60-mm measurement distally was performed to the cortical edge of the tibia. The first set of points was marked on the cortical edges for both the medial and lateral sides. The second set of points was measured 30 mm distally from the first set of points (90 mm from the proximal-most portion of the tibia). Bisecting these two sets of points created the tibial line. Extending the femoral line distally and tibial line proximally into the joint space to create an intersection point allowed the angle measurement tools in Synapse to measure the angle between the femoral line and the tibial line. The same measurements were taken to establish postoperative tibiofemoral angles. Negative values were considered varus, and positive values were considered valgus. Preoperative tibiofemoral angle was subtracted from postoperative tibiofemoral angle to identify the amount of change (delta) from preoperative to postoperative alignment in degrees.

### Data Analysis

All statistical analyses were conducted in Minitab 18. Outliers were assessed with the Dixon r22 ratio test due to the size of the study groups. Because of kinematic differences of knees based on posterior cruciate ligament (PCL) disposition, implant design, and the performed activity (ie, early flexion versus late flexion),^7,8,25,26,27,28,29,30^ univariate and then multivariate analyses were conducted to identify predictors of pivot patterns within PCL disposition (resected or fully intact) and flexion angle groups (early flexion, 0° to 45°; midflexion, 45° to 90°; and late flexion, 90° maximum). Univariate continuous variables of two groups were compared with an unpaired two-sample (t) Student *t*-test. Univariate categorical variables were compared with a chi-square test, with Fisher *P* reported for 2 × 2 contingency tables. All univariate analyses with *P* ≤ 0.200 were entered into a binary logistic regression (BLR) model with lateral pivot defined as the outcome. A stepwise backward elimination method was used to remove insignificant model variables. A significance level of 0.05 was used for the final models.

## Results

**Figure 3:**
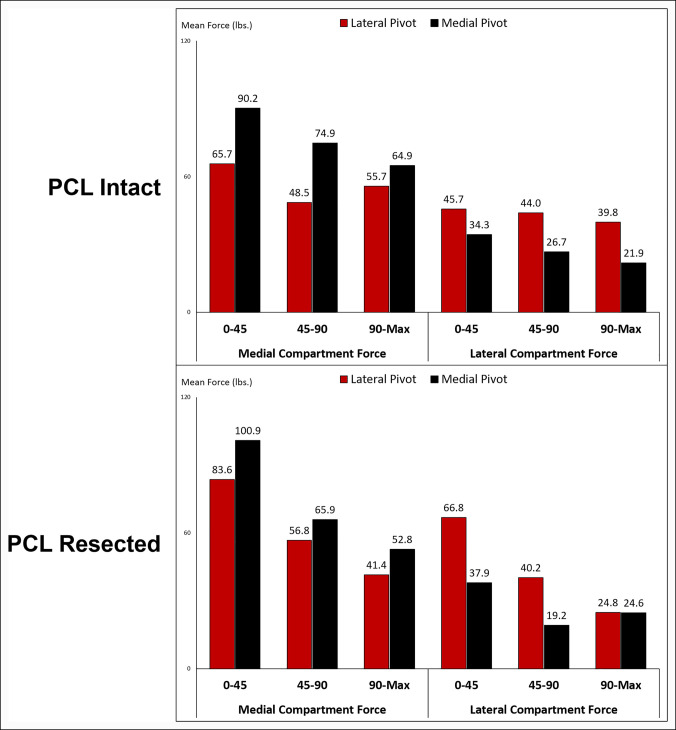
TKAs classified as a medial pivot demonstrated higher medial compartment forces, whereas TKAs classified as a lateral pivot demonstrated higher lateral compartment forces at all flexion arcs regardless of PCL disposition. PCL = posterior cruciate ligament, TKA = total knee arthroplasty

One hundred fifty-seven TKAs were available for analysis. The mean age was 63.2 years (SD 9.9, range 38.8 to 88.0 years), and the mean body mass index (BMI) was 33.7 kg/m^2^ (SD, 7.6; range 18.0 to 59.7 kg/m^2^). Seventy-five percent of the cohort was female. The mean preoperative tibiofemoral angle was 0.7° (SD, 6.2; range −17° to 20.0°), the mean postoperative tibiofemoral angle was 3.8° (SD, 2.2; range −1.0° to 11.0°), and the mean change in tibiofemoral angle was 3.1° (SD, 6.4; range −18.0° to 20.0°). The PCL was fully resected in 59% of the cohort, whereas the remaining 41% had a fully intact PCL during data collection. Knees classified as central or translating pivots in early flexion (20), midflexion (16), and late flexion (11) and were removed from analysis to isolate the effect of medial vs. lateral pivot knees. In early flexion, 59% (81/137) of knees were classified as a lateral pivot (n = 23 and 58 for the intact PCL and resected PCL groups, respectively). In midflexion, 50% (71/141) of knees were classified as a lateral pivot (n = 25 and 46 for the intact PCL and resected PCL groups, respectively). In late flexion, 60% (87/145) of knees were classified as a lateral pivot (n = 26 and 61 for the intact PCL and resected PCL groups, respectively). Compartmental forces by pivot pattern and PCL disposition are characterized in Figure 3.

### Early flexion (0° to 45°)—Intact Posterior Cruciate Ligament

In early flexion with a fully intact PCL, 41.1% of patients were classified as a lateral pivot knee (n = 23), whereas 58.9% were classified as a medial pivot knee (n = 33). Covariates included in the BLR model were BMI, medial compartment force, the force difference between the two compartments, and postoperative tibiofemoral angle (Supplemental Table S1, http://links.lww.com/JG9/A205, *P* ≤ 0.185). However, none of these variables were significant predictors of a lateral pivot in early flexion with a retained PCL.

### Early flexion (0° to 45°)—Resected Posterior Cruciate Ligament

In early flexion with a fully resected PCL, 71.6% of patients were classified as a lateral pivot knee (n = 58), whereas 28.4% were classified as a medial pivot knee (n = 23). Covariates included in the BLR model were lateral compartment force, force difference between the two compartments, and preoperative tibiofemoral angle (Supplemental Table S2A, http://links.lww.com/JG9/A206, *P* ≤ 0.187). However, only lateral compartment force was a significant predictor of a lateral pivot with a fully resected PCL (Supplemental Table S2B, http://links.lww.com/JG9/A206, *P* = 0.031). When the PCL was resected, the BLR model predicted an increasing chance of a lateral pivot in early flexion as lateral compartment force increased (Supplemental Table S2B, http://links.lww.com/JG9/A206; odds ratio [OR], 1.5).

### Midflexion (45° to 90°)—Intact Posterior Cruciate Ligament

In midflexion with a fully intact PCL, 41.7% of patients were classified as a lateral pivot knee (n = 25), whereas 58.3% were classified as a medial pivot knee (n = 35). Covariates included in the BLR model were BMI, medial compartment force, lateral compartment force, the force difference between the two compartments, postoperative tibiofemoral alignment, and delta tibiofemoral alignment (Supplemental Table S3A, http://links.lww.com/JG9/A207, *P* ≤ 0.098). However, only a decreasing medial compartment force was a significant predictor of a lateral pivot with an intact PCL (Supplemental Table S3B, http://links.lww.com/JG9/A207, *P* = 0.037). The inverse of this finding also was valid—increasing medial compartment force was predictive of a medial pivot outcome. BMI was included in the final model for a better model fit; however, the confidence interval of the odds ratio included the null hypothesis (1.0); therefore, it was not a statistically significant predictor of a lateral pivot (Supplemental Table S3B, http://links.lww.com/JG9/A207, 95% CI, 0.98 to 2.16). When the PCL was retained, the BLR model predicted an increasing chance of a lateral pivot in midflexion as medial compartment force decreased (Supplemental Table S3B, http://links.lww.com/JG9/A207, OR, 0.71).

### Midflexion (45° to 90°)—Resected Posterior Cruciate Ligament

In midflexion with a fully resected PCL, 56.8% of patients were classified as a lateral pivot knee (n = 46), whereas 43.2% were classified as a medial pivot knee (n = 35). Covariates included in the BLR model were lateral compartment force and force difference between the two compartments (Supplemental Table S4A, http://links.lww.com/JG9/A208, *P* ≤ 0.053). However, only lateral compartment force was a significant predictor of a lateral pivot with a fully resected PCL (Supplemental Table S4B, http://links.lww.com/JG9/A208, *P* = 0.018). When the PCL was resected, the BLR model predicted an increasing chance of a lateral pivot in midflexion as the lateral compartment force increased (Supplemental Table S4B, http://links.lww.com/JG9/A208, OR, 1.76).

### Late flexion (90° to Maximum Flexion)—Intact Posterior Cruciate Ligament

In late flexion with a fully intact PCL, 44.1% of patients were classified as a lateral pivot knee (n = 26), whereas 55.9% were classified as a medial pivot knee (n = 33). Covariates included in the BLR model were lateral compartment force and force difference between the two compartments (Supplemental Table S5A, http://links.lww.com/JG9/A209, *P* ≤ 0.097). However, only lateral compartment force was a significant predictor of a lateral pivot with an intact PCL (Supplemental Table S5B, http://links.lww.com/JG9/A209, *P* = 0.026). With the PCL retained, the BLR model predicted an increasing chance of a lateral pivot in late flexion as the lateral compartment force increased (Supplemental Table S5B, http://links.lww.com/JG9/A209, OR 1.87).

### Late flexion (90° to Maximum Flexion)—Resected Posterior Cruciate Ligament

In late flexion with a fully resected PCL, 70.9% of patients were classified as a lateral pivot knee (n = 61), whereas 29.1% were classified as a medial pivot knee (n = 25). BMI was the only covariate included in the BLR model (Supplemental Table S6A, http://links.lww.com/JG9/A210, *P* = 0.046); however, the confidence interval of the OR included the null hypothesis (1.0); therefore, it was not a statistically significant predictor of a lateral pivot (Supplemental Table S6B, http://links.lww.com/JG9/A210; 95% CI, 0.95 to 2.02). No variables were significant predictors of a lateral pivot in late flexion with a resected PCL. A summary of all multivariate model main effects is shown in Table 1.

**Table 1:** BLR Result Summary—Predictors of Pivot Pattern

	Early Flexion 0°-45°	Midflexion 45°-90°	Late Flexion 90°-Maximum
PCL-intact TKAs	None	Increased medial force = medial pivot, OR 1.4, *P* = 0.037	Increased lateral force = lateral pivot, OR 1.9, *P* = 0.026
PCL-resected TKAs	Increased lateral force = lateral pivot, OR 1.5, *P* = 0.031	Increased lateral force = lateral pivot, OR 1.8, *P* = 0.018	None

BLR = binary logistic regression, OR = odds ratio, PCL = posterior cruciate ligament, TKA = total knee arthroplasty

## Discussion

There was sufficient evidence to reject the null hypothesis of the current study. Study results show that at certain flexion ranges, kinematic pivot patterns were predictable based on the amount of intraoperative compartment contact force via soft-tissue balance in combination with PCL disposition. No predictors of a lateral pivot were identified with an intact PCL in early flexion (when the PCL is not engaged), but as the knee increased in flexion and the PCL engages, the increase of lateral force in late flexion was a significant predictor of a lateral pivot (Table 1). Interestingly, for intact PCL knees at midflexion, the increase of medial force was a predictor of a medial pivot pattern. A higher medial force predicting a medial pivot in midflexion and higher lateral force predicting a lateral pivot in late flexion in intact PCL knees could be due, in part, to soft-tissue interference of patients with high BMI, the parabolic behavior of PCL elongation, as it becomes increasingly elongated up to midflexion and then decreases beyond maximum flexion (>120°).^31^ Other reasons explaining this finding could be interplay between the complex movements of twisting and angle changes of the PCL^32^ and force distribution changes^33^ during increased flexion along with high variability of PCL shape, size, and femoral attachment locations.^34^ Conversely, in PCL resected knees, an increase in lateral force predicted a lateral pivot pattern in both early and midflexion, however, not in late flexion (Table 1). We hypothesize within the setting of PCL resected knees, it provided a more controlled evaluation of the contact force effect on pivot patterns as the variability of PCL shape, size, and femoral attachment location was eliminated by resection of the PCL. The lack of cruciate ligaments in these analyses allowed the direct evaluation of contact force on pivot pattern in a nonconforming polyethylene insert, showing that when the PCL is resected, an increased lateral compartment force in early and midflexion led to a lateral pivot pattern.

Kinematic pivot patterns in knees have been studied extensively^4,5,6,7,8^; however, few studies investigate the interaction of kinematic pivot patterns and tibiofemoral compartment forces. Wasielewski et al found intraoperative compartmental imbalance to be associated with inappropriate kinematics via fluoroscopy and increased condylar lift-off proving that surgical technique influences the force distribution between the compartments and subsequent postoperative kinematics.^35,36^ However, very few studies exist to define the limits of a well-balanced knee. Most importantly, there are even fewer studies that determine whether condylar lift-off observed in fluoroscopic studies correlates with suboptimal clinical function. Varadarajan et al^37^ used novel instrumented tibial implants to measure in vivo tibiofemoral articular contact forces and contact kinematics in three subjects during dynamic activities, which found articular contact forces and distributions to be both patient and activity specific. In addition, Dennis et al^5^ published a comprehensive kinematic analysis of 811 TKAs of numerous designs, institutions, and surgeons and reported substantial variability among all designs and kinematic patterns. The authors reported that a desirable medial pivot pattern in flexion was present in only 55% of TKAs, suggesting that surgeons have little ability to reliably induce a particular kinematic pivot pattern in TKA. Recently, Young et al^38^ report on intraoperative kinematic patterns using principal component analysis and extracted four frontal plane phenotypic kinematic patterns, which explained 99.9% of the variability. Although these four frontal plane patterns explained essentially all the kinematic pattern variability using modern implants, the ability to consistently induce an ideal target kinematic pattern surgically with a nonconforming polyethylene insert remains unknown and highly variable. Furthermore, these studies (like the current study) focus on only one or two planes of motion but do not account for motion in other planes, which could significantly affect optimized knee motion leading to improvement in patient outcomes.

This study should be considered in the context of limitations primarily related to the intraoperative condition of data collection. The translation of these intraoperative contact forces and patterns to postoperative, weight-bearing kinematics is unknown and warrants further research at varying flexion angles. In addition, high SDs in compartment force were observed, which calls into question the accuracy and precision of the sensor-embedded compartment force-measuring device. Furthermore, this study only evaluated axial kinematic pivot patterns and did not account for sagittal alignment or motion in other planes.

## Conclusion

This is one of the first studies correlating tibiofemoral compartment forces with kinematic pivot patterns observed intraoperatively. These results suggest that intraoperative pivot patterns can be predicted based on the amount of intraoperative tibiofemoral compartment contact force during TKA. Further research is recommended to confirm that intraoperative kinematic pivot patterns correlate with in vivo, weight-bearing, postoperative kinematics, and clinical outcomes. These data could benefit surgeons who want to facilitate a certain pivot pattern based on intraoperative gap balancing of the knee.
